# Effects of Tai Chi on functional performance and fall-related psychological outcomes in Parkinson’s disease: a systematic review and meta-analysis

**DOI:** 10.3389/fnagi.2026.1860410

**Published:** 2026-08-14

**Authors:** Fusen Wang, Tongtong Hao

**Affiliations:** Beijing Sport University, Beijing, China

**Keywords:** intervention characteristics, meta-analysis, motor performance, Parkinson’s disease, psychosocial well-being, Tai Chi

## Abstract

**Objective:**

To systematically evaluate the effects of Tai Chi on objective motor performance and subjective fall-related psychological outcomes in patients with Parkinson’s disease (PD), and to examine whether intervention characteristics influence the magnitude of therapeutic effects.

**Methods:**

Randomized controlled trials (RCTs) investigating Tai Chi interventions in patients with PD were identified through searches of electronic databases from inception to April 2026. The methodological quality and risk of bias were assessed using the Cochrane Risk of Bias 2 (RoB 2) tool and the PEDro scale. Meta-analyses were performed to evaluate the effects of Tai Chi on balance function, motor function, mobility, balance confidence, and fear of falling. Subgroup analyses were conducted according to intervention duration, weekly frequency, and session length to explore potential sources of heterogeneity.

**Results:**

Seventeen RCTs involving 914 participants were included. Meta-analysis demonstrated that Tai Chi significantly improved balance function measured by the Berg Balance Scale (BBS) (MD = 3.35, 95% CI [1.40, 5.30], *p* < 0.001), motor function assessed by the Unified Parkinson’s Disease Rating Scale Part III (UPDRS-III) (MD = −4.10, 95% CI [−6.34, −1.85], *p* < 0.001), and mobility measured by the Timed Up and Go Test (TUGT) (MD = −1.97, 95% CI [−3.30, −0.64], *p* < 0.001). Limited evidence suggested possible improvements in fall-related psychological outcomes, including balance confidence and fear of falling; however, these findings should be interpreted cautiously due to the small number of studies, heterogeneity, and limited precision.

**Conclusion:**

Tai Chi may improve balance, motor function, and mobility in patients with Parkinson’s disease. Evidence regarding fall-related psychological outcomes remains limited and requires further investigation. However, the optimal intervention dosage remains unclear, and the findings should be interpreted cautiously due to heterogeneity among studies and limited evidence for subjective outcomes. Future large-scale, well-designed RCTs with standardized intervention protocols and longer follow-up periods are warranted.

**Systematic review registration:**

https://www.crd.york.ac.uk/PROSPERO/view/CRD420261371910, identifier CRD420261371910.

## 1 Introduction

Parkinson’s disease (PD) represents a growing global health challenge associated with population aging and increasing disease burden. Epidemiological studies have demonstrated a substantial increase in the number of individuals affected by PD worldwide over recent decades, making it one of the fastest-growing neurological disorders (Dorsey et al., 2020). The rising prevalence of PD has contributed considerably to disability burden, mortality, and healthcare demands, particularly due to progressive motor impairment, falls, and loss of functional independence. Parkinson’s disease is characterized by progressive motor impairments, including bradykinesia, rigidity, resting tremor, and postural instability (Armstrong and Okun, 2020). As the disease progresses, gait disturbances and impaired postural control substantially increase the risk of falls, restrict functional independence, and negatively affect quality of life (Morris et al., 2021).

Pharmacological therapy, particularly dopaminergic medication, remains the cornerstone of PD management (Kalia and Lang, 2015). However, medication-related limitations, including incomplete control of postural instability, gait dysfunction, and non-motor complications, highlight the importance of complementary non-pharmacological interventions. Exercise-based rehabilitation has increasingly been recognized as an essential component of comprehensive PD management due to its potential to improve motor performance, maintain functional capacity, and enhance psychological well-being (Bloem et al., 2021).

Tai Chi, a traditional mind–body exercise characterized by slow controlled movements, continuous weight shifting, postural transitions, and cognitive-motor integration, has attracted increasing attention as a rehabilitation strategy for individuals with PD (Hao et al., 2026b). Unlike conventional aerobic or resistance exercise, Tai Chi simultaneously challenges balance control, lower-limb strength, movement coordination, and attentional regulation (Hao et al., 2026b). These characteristics may provide potential benefits for improving postural stability, gait performance, and confidence in movement-related activities (Xu et al., 2026). Previous randomized controlled trials (RCTs) have reported beneficial effects of Tai Chi on outcomes such as balance function, motor symptoms, mobility, and fall-related psychological measures in patients with PD.

However, several important uncertainties remain. First, previous systematic reviews and meta-analyses have primarily focused on isolated physical outcomes, such as balance or motor symptoms, while the relationship between objective functional improvements and subjective experiences related to falls has not been comprehensively examined (Yu et al., 2021). Since fall risk in PD is influenced not only by impaired physical performance but also by psychological factors such as fear of falling and reduced balance confidence, an integrated evaluation of both dimensions is clinically meaningful (Liu et al., 2019). Second, considerable variability exists among Tai Chi interventions, including differences in training duration, weekly frequency, session length, Tai Chi styles, and participant characteristics. Although intervention dosage has been proposed as a potential determinant of exercise effectiveness, whether these parameters modify the therapeutic effects of Tai Chi in PD remains unclear (Xie et al., 2022). Third, existing studies vary in methodological quality, sample size, and outcome assessment methods, limiting the strength and generalizability of current conclusions.

Therefore, this systematic review and meta-analysis aimed to comprehensively evaluate the effects of Tai Chi on both objective functional outcomes and subjective fall-related psychological outcomes in patients with Parkinson’s disease. Specifically, this study assessed the effects of Tai Chi on balance function, motor symptoms, mobility, balance confidence, and fear of falling. In addition, subgroup analyses were performed according to intervention duration, weekly frequency, and session length to explore whether commonly reported training characteristics influenced the magnitude of treatment effects. By integrating physical and psychological outcomes, this study aims to provide a more comprehensive understanding of the potential role of Tai Chi as a complementary rehabilitation approach for individuals with PD.

## 2 Methods

### 2.1 Registration and procedural protocol

This systematic review and meta-analysis was conducted in accordance with the Preferred Reporting Items for Systematic Reviews and Meta-Analyses (PRISMA) 2020 statement (Page et al., 2021). The review protocol was registered in PROSPERO (CRD420261371910) on April 17, 2026. The predefined procedures, including study selection, data extraction, risk of bias assessment, and statistical analysis, were followed throughout the review process, and no major deviations occurred. The registered protocol was entitled “From Gait to Confidence: A Meta-Analysis of Tai Chi on Objective and Subjective Physical and Mental Outcomes in Parkinson’s Disease.” The predefined procedures, including literature searching, study selection, data extraction, risk of bias assessment, and statistical analysis, were followed throughout the review process.

### 2.2 Search strategy

Our systematic search, finalized on April 10, 2026, utilized structured MeSH terms and varied keywords to ensure maximum coverage. We scoured both global and domestic sources–namely PubMed, Embase, Cochrane Library, Web of Science, CNKI, Wanfang, VIP, and CBM–incorporating all available literature since database inception. The primary search terms included: (1) Disease-related terms: “Parkinson’s disease,” “Parkinsonism,” and “PD”; (2) Intervention-related terms: “Tai Chi,” “Tai Ji Quan,” “Tai Chi Chuan,” “Tai Ji,” “Yang-style Tai Chi,” “Chen-style Tai Chi,” and “Qigong”; and (3) Outcome-related terms: “gait,” “balance,” “fall,” “Unified Parkinson’s Disease Rating Scale (UPDRS),” “Berg Balance Scale (BBS),” “Timed Up and Go Test (TUGT),” “Activities-specific Balance Confidence scale (ABC),” and “Modified Falls Efficacy Scale (MFES).” Comprehensive search strategies were developed for each database according to its specific indexing terms and search functions. The detailed search strategy for the PubMed database is presented in Table 1.

**TABLE 1:** Example of search strategy for the PubMed database.

Number	Search terms
#1	Parkinson’s disease
#2	Parkinson
#3	PD
#4	#1 OR #2 OR #3
#5	Tai Chi
#6	Tai Ji Quan
#7	Qigong
#8	#5 OR #6 OR #7
#9	Gait
#10	Balance
#11	Fall
#12	UPDRS OR BBS OR TUGT
#13	#9 OR #10 OR #11 OR #12
#14	Randomized controlled trial
#15	RCT
#16	#14 OR #15
#17	#4 AND #8 AND #13 AND #16

### 2.3 Inclusion and exclusion criteria

The eligibility criteria for literature selection were established based on the PICOS framework (Population, Intervention, Comparison, Outcome, and Study design) (Higgins et al., 2024). Detailed inclusion and exclusion criteria, along with specific outcome measures, are presented in Table 2.

**TABLE 2:** Inclusion and exclusion criteria.

Parameter	Inclusion criteria	Exclusion criteria
P (participants)	Adults (≥18 years) clinically diagnosed with idiopathic Parkinson’s disease (e.g., UK Parkinson’s Disease Society Brain Bank criteria); regardless of gender, disease duration, or Hoehn-Yahr stage.	Secondary parkinsonism (e.g., drug-induced, vascular, or post-encephalitic); comorbidities with severe cardiac, hepatic, or renal dysfunction; cognitive impairment hindering training cooperation; pregnant or lactating women.
I (intervention)	Tai Chi (all styles, e.g., Yang, Chen, Wu, Sun; and simplified forms, e.g., 8-form, 24-form); must provide detailed records of frequency, session duration, and total duration.	Studies lacking specific exercise dosage; Tai Chi combined with other interventions where its independent effect cannot be isolated; acute (single-session) interventions; or uncontrolled trials.
C (comparison)	Control groups receiving routine medication, conventional rehabilitation, health education, waiting list, no intervention, or non-Tai Chi exercises (e.g., walking, stretching, conventional balance training).	Uncontrolled studies; control groups receiving Tai Chi or interventions containing Tai Chi components.
O (outcomes)	Core objective metrics: motor function (e.g., UPDRS-III), balance (e.g., BBS), and mobility (e.g., TUGT); core subjective metrics: balance confidence (e.g., ABC) and fear of falling (e.g., MFES).	Studies with unavailable raw data or failure to report required outcomes; studies reporting only qualitative results without quantitative data.
S (setting/study design)	Peer-reviewed randomized controlled trials (RCTs); restricted to English and Chinese languages.	Reviews, systematic reviews, case reports, conference abstracts, non-academic reports, and duplicate publications.

### 2.4 Literature selection and data extraction

Fusen Wang and Tongtong Hao independently performed the study screening and data extraction. The consistency of the results was verified through cross-checking, and any disagreements were resolved through discussion or consultation with a third senior researcher to reach consensus (Higgins et al., 2024).

We identified eligible trials by discarding unsuitable studies during a preliminary title-abstract scan, after which each potential candidate underwent a detailed full-text review. Where data were insufficient or poorly defined, proactive efforts were made to contact the study authors for data recovery.

The extracted data elements comprised: (1) Bibliographic info (title, author, year, and region); (2) Participant baseline (size, age, gender, and PD severity); (3) Exercise parameters (style, duration, and frequency); (4) Bias assessment factors; and (5) Outcome statistics (Means/SDs for all analyzed scales including UPDRS-III, BBS, TUGT, ABC, and MFES).

### 2.5 Risk of bias assessment

Using an independent peer-review model, two authors appraised the study quality, with any unsettled conflicts resolved by the intervention of a third expert (Sterne et al., 2019). Each trial’s bias risk was appraised via the Revised Cochrane Risk of Bias tool (RoB 2). This assessment encompassed five distinct domains, wherein we scrutinized the randomization process, deviations from assigned protocols, the presence of missing data, the reliability of outcome measurements, and potential biases in result selection (Wang et al., 2025). Methodological quality was additionally appraised via the PEDro scale, where a cumulative score of up to 10 is calculated. This score excludes the initial item and is based exclusively on the assessment of criteria 2 through 11 within the 11-item checklist. According to the PEDro criteria, study quality was categorized as: high quality (≥6 points), fair quality (4–5 points), and poor quality (≤3 points) (Maher et al., 2003).

### 2.6 Statistical analysis

We leveraged SPSSAU software to perform the statistical synthesis, where continuous data effects were defined as MD or SMD (Hedges’ g). Precision of these estimates was further substantiated by calculating 95% confidence intervals (CIs) (Suero et al., 2025). MD was utilized when studies employed identical measurement tools and units for the same outcome; otherwise, SMD was adopted (Rücker et al., 2025).

Heterogeneity across studies was quantified using the I^2^ statistic and Cochran’s Q test. A fixed-effects model was employed if heterogeneity was low (I^2^ < 50% and *p* > 0.1 for the Q test). Conversely, if significant heterogeneity was observed (I^2^ ≥ 50% or *P* ≤ 0.1), a random-effects model was implemented (Riley et al., 2011).

We partitioned the data to examine the moderating role of intervention intensity, including duration, frequency, and session length. The significance of differences between these predefined strata was determined using the Q test to ensure a robust exploration of heterogeneity (Huang et al., 2025).

Publication bias was assessed only for outcomes with at least 10 included studies, as recommended for reliable interpretation of funnel plots and Egger’s regression tests (Egger et al., 1997). Egger’s regression test and funnel plot asymmetry were performed when sufficient numbers of studies were available. Findings were considered statistically significant if two-tailed tests yielded *p* < 0.05, with the alpha level strictly set at 0.05 (Altman and Bland, 1995).

## 3 Results

### 3.1 Literature selection process

**FIGURE 1:**
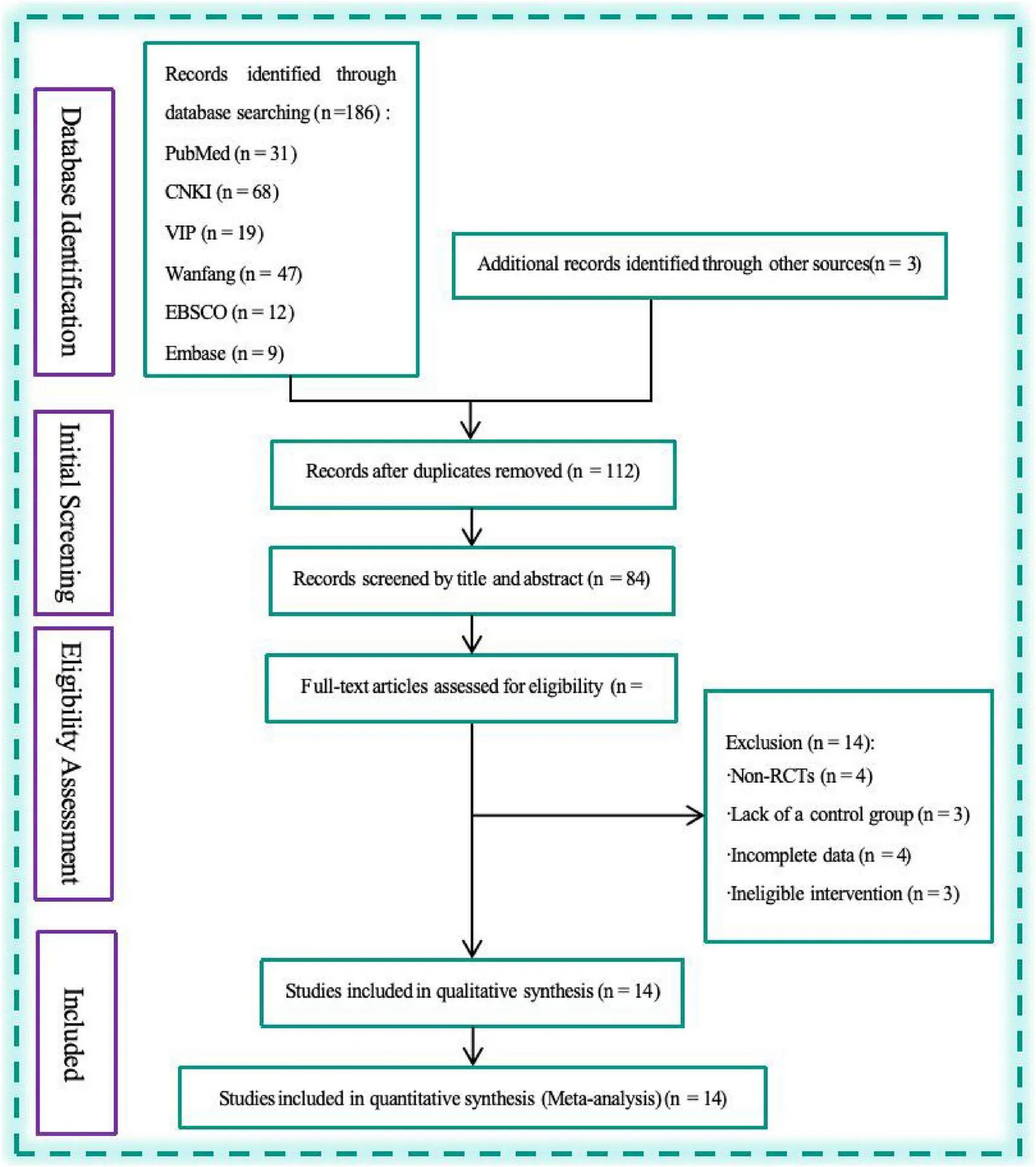
Literature screening process and results.

The literature selection process is presented in the PRISMA flow diagram (Figure 1). A total of 189 records were initially identified, including 186 records retrieved from electronic databases and 3 additional records obtained from other sources. After removing duplicates, 112 records remained for screening. Following title and abstract screening, 84 records were excluded because they did not meet the predefined eligibility criteria. The full texts of 28 potentially relevant articles were assessed for eligibility. Among these, 14 studies were excluded due to reasons including non-randomized controlled trial design (*n* = 4), lack of a control group (*n* = 3), incomplete data reporting (*n* = 4), and ineligible intervention protocols (*n* = 3). Finally, 14 randomized controlled trials were included in the qualitative synthesis and quantitative meta-analysis.

### 3.2 Characteristics of included studies

The characteristics of the included randomized controlled trials are summarized in Table 3. A total of 14 RCTs involving patients with Parkinson’s disease were included, with publication years ranging from 2011 to 2025. All studies were conducted in China, and the sample sizes ranged from 16 to 81 participants. The included participants were diagnosed with Parkinson’s disease according to the diagnostic criteria reported in each original study, and most trials recruited patients with mild-to-moderate disease severity. The intervention groups received various forms of Tai Chi, including Yang-style Tai Chi and other modified Tai Chi protocols, whereas control groups mainly received conventional rehabilitation, routine care, health education, or other non-Tai Chi interventions.

**TABLE 3:** Baseline profiles and intervention protocols of the selected trials.

References	Year	Mean age (I/C, years)		Sample size (I/C, *n*)		Intervention description (I/C)		Intervention dosage	Outcome measures	Follow-up
		Intervention group	Control group	Intervention group	Control group	Intervention group	Control group			
Yang et al., 2025	2025	58.22 ± 5.47	59.27 ± 7.50	32	30	24-form Tai Chi + basic PD treatment vs.	Basic PD treatment, maintaining habitual lifestyle	12W-3/week-60 min	UPDRS-III, BBS, TUGT, MFES	Weekly telephone follow-up
Guan et al., 2018	2018	69.46 ± 5.45	68.61 ± 6.22	41	40	24-form Tai Chi + basic PD treatment vs.	Basic PD treatment, maintaining habitual lifestyle	24W-4/week-60 min	BBS, TUGT, ABC	None
Lu, 2017	2017	67.75 ± 6.84	68.20 ± 7.32	8	8	24-form Tai Chi + Madopar vs.	Madopar only	8W-5/week-60 min	UPDRS-III, BBS	None
Guan et al., 2016	2016	70.23 ± 4.24	69.71 ± 4.13	31	31	24-form Tai Chi + routine rehabilitation vs.	Routine neuro-rehabilitation	12W-4/week-60 min	BBS, TUGT, ABC	None
Wang et al., 2023	2023	69.80 ± 6.90	67.13 ± 8.33	15	15	24-form Tai Chi vs.	No intervention	24W-3/week-60 min	UPDRS-III, BBS, TUGT	6-months follow-up
Xiao et al., 2021	2021	72.78 ± 2.63	72.58 ± 2.62	20	20	24-form Tai Chi + routine medication vs.	Routine medication + exercise guidance (walking, balance, posture)	26W-4/week-60 min	BBS, ABC	None
Li L. et al., 2017	2017	65.25 ± 6.37	67.78 ± 5.36	42	38	Adapted 8-form Yang-style Tai Chi + medication vs.	Medication only	16W-3/week-60 min	UPDRS-III	Weekly hospital follow-up
Ji et al., 2016	2016	56.06 ± 11.16	59.13 ± 11.22	19	19	Simplified 8-form Chen-style Tai Chi + Levodopa & DA agonists vs.	Levodopa & DA agonists only	13W-7/week-60 min	UPDRS-III, BBS	None
Chen et al., 2016	2017	68.1 ± 8.2	68.2 ± 8.1	15	15	“Self-developed Tai Chi” + medication vs.	Routine medication + conventional physiotherapy	8W-7/week-60 min	BBS	None
Yi et al., 2011	2011	63.35 ± 8.72	64.83 ± 9.29	20	20	24-form Tai Chi + Madopar vs.	Walking exercise + Madopar	4W-2/day-45 min	UPDRS-III, BBS	None
Guan et al., 2017	2017	>60	>60	40	40	24-form Tai Chi + Madopar vs.	Walking exercise + Madopar	24W-5/week-60 min	BBS, MFES	None
Wang et al., 2016	2016	67.6 ± 8.4	68.0 ± 8.5	40	40	24-form Tai Chi + Amitriptyline vs.	Amitriptyline only	16W-2/day-60 min	UPDRS-III, BBS	None
Li, 2011	2011	68.28 ± 6.62	67.13 ± 6.73	24	23	24-form Tai Chi + Madopar vs.	Walking exercise + Madopar	8W-2/day-45 min	UPDRS-III, BBS	None
Li B. et al., 2017	2017	≥60	≥60	30	30	24-form Tai Chi + routine nursing care vs.	Routine neuro-nursing care	12W-4/week-60 min	BBS, MFES	None

IG, intervention group; CG, control group; W, weeks; min, minutes; DA, dopamine; UPDRS-III, Unified Parkinson’s Disease Rating Scale Part III; BBS, Berg Balance Scale; TUGT, Timed Up and Go Test; ABC, Activities-specific Balance Confidence Scale; MFES, Modified Falls Efficacy Scale.

The intervention characteristics varied across studies. The intervention duration ranged from 4 to 26 weeks. Session duration varied from 46 to 60 min. The primary outcomes included objective motor and balance-related measures, such as the Berg Balance Scale (BBS), Unified Parkinson’s Disease Rating Scale Part III (UPDRS-III), and Timed Up and Go Test (TUGT), as well as subjective outcomes including balance confidence and fear of falling. Due to variations in intervention protocols and outcome assessments among studies, subgroup analyses were conducted based on predefined intervention characteristics, including duration, weekly frequency, and session length, to explore potential sources of heterogeneity.

### 3.3 Risk of bias and quality assessment

**FIGURE 2:**
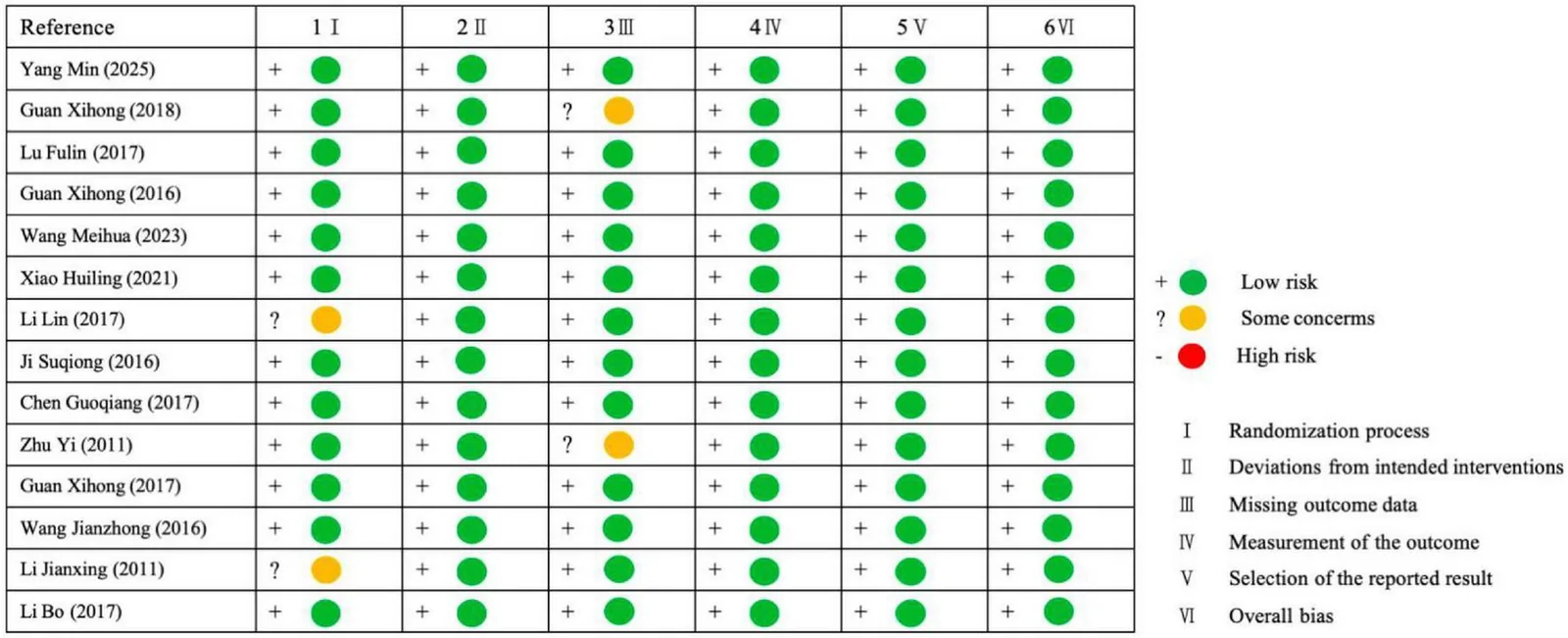
Methodological bias profile of the selected RCTs as appraised by the Cochrane RoB 2 framework.

The risk of bias of the included randomized controlled trials was assessed using the Cochrane Risk of Bias 2 (RoB 2) tool, and methodological quality was evaluated using the PEDro scale (Figure 2 and Table 4). Overall, the included studies demonstrated generally acceptable methodological quality; however, several methodological concerns were identified. In particular, incomplete reporting of allocation concealment, limited information regarding assessor blinding, and the inherent difficulty of blinding participants and therapists in exercise-based interventions may have introduced potential sources of bias.

**TABLE 4:** Comprehensive breakdown of bias risk categories for each synthesized trial according to RoB 2 criteria.

References	PEDro scale											
	①	②	③	④	⑤	⑥	⑦	⑧	⑨	⑩	⑪	Score
Yang et al., 2025	Yes	1		1	1			1	1	1	1	7
Guan et al., 2018	Yes	1		1				1	1	1	1	6
Lu, 2017	Yes	1		1				1	1	1	1	6
Guan et al., 2016	Yes	1		1			1	1	1	1	1	7
Wang et al., 2023	Yes	1		1				1	1	1	1	6
Xiao et al., 2021	Yes	1		1				1	1	1	1	6
Li L. et al., 2017	Yes	1		1				1	1	1	1	6
Ji et al., 2016	Yes	1		1			1	1	1	1	1	7
Chen et al., 2016	Yes	1		1				1	1	1	1	6
Yi et al., 2011	Yes	1		1				1	1	1	1	6
Guan et al., 2017	Yes	1		1				1	1	1	1	6
Wang et al., 2016	Yes	1		1				1	1	1	1	6
Li, 2011	Yes	1		1				1	1	1	1	6
Li B. et al., 2017	Yes	1		1				1	1	1	1	6

① Selection criteria were clearly stated; ② Subjects were randomized; ③ Hidden allocation process; ④ Initial similarity in key prognostic factors; ⑤ Masking of participants; ⑥ Masking of providers; ⑦ Masking of outcome evaluators; ⑧ Completion of follow-up by ≥85% of sample; ⑨ Analysis by original assigned groups (ITT); ⑩ Results of between-group tests; ⑪ Inclusion of effect size means and variability.

Although most studies adequately addressed randomization, outcome reporting, and missing outcome data, these limitations should be considered when interpreting the pooled findings. The PEDro scores ranged from 6 to 7, indicating moderate-to-high methodological quality. However, methodological quality does not necessarily eliminate the risk of bias, and the certainty of evidence should therefore be interpreted cautiously.

The certainty of evidence for the main outcomes was evaluated using the GRADE framework (Table 5). The certainty of evidence ranged from very low to low across outcomes. The evidence for balance function (BBS), motor function (UPDRS-III), and mobility (TUGT) was rated as low certainty, mainly due to substantial heterogeneity among studies, methodological limitations, and imprecision of effect estimates. The certainty of evidence for balance confidence (ABC) and fear of falling (MFES/FES) was rated as very low because of the limited number of available studies, wide confidence intervals, and concerns regarding inconsistency. Therefore, although pooled analyses suggested potential benefits of Tai Chi, the overall certainty of evidence remains limited.

**TABLE 5:** Certainty of evidence assessment for main outcomes according to the GRADE approach.

Outcome	Certainty
BBS	Low
UPDRS-III	Low
TUGT	Low
ABC	Very low
MFES	Very low

### 3.4 Effect of Tai Chi on balance function (BBS)

**FIGURE 3:**
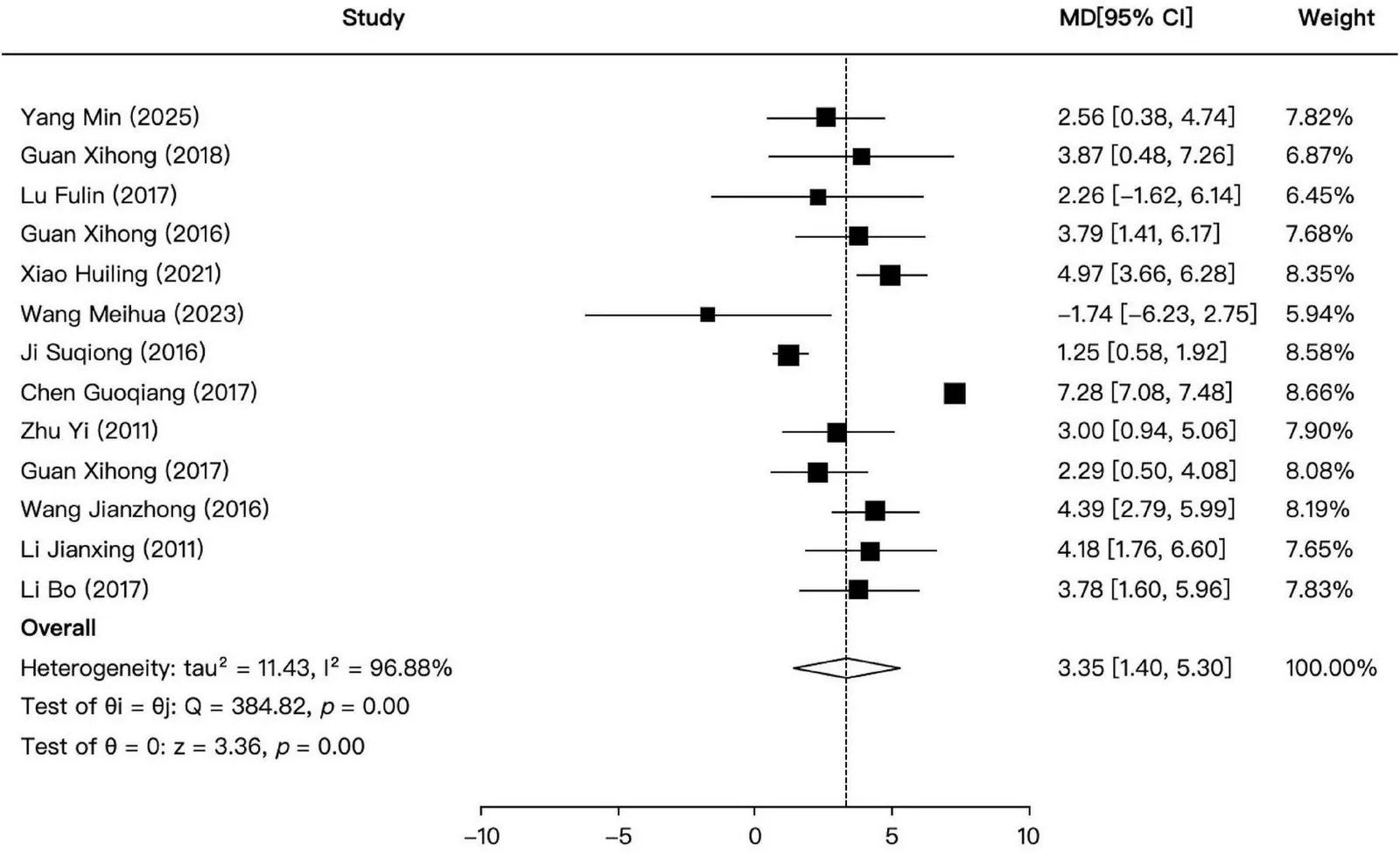
Forest plot delineating the therapeutic efficacy of Tai Chi interventions on postural stability (measured by BBS) in the Parkinson’s population.

A total of 13 randomized controlled trials involving patients with Parkinson’s disease were included to evaluate the effects of Tai Chi on balance function measured by the Berg Balance Scale (BBS). As shown in Figure 3, the pooled analysis using a random-effects model demonstrated that Tai Chi significantly improved BBS scores compared with control conditions (MD = 3.35, 95% CI [1.40, 5.30], *z* = 3.36, *p* < 0.001). This indicates that Tai Chi interventions were associated with a significant improvement in balance function among patients with Parkinson’s disease. However, substantial heterogeneity was observed among the included studies (τ^2^ = 11.43, I^2^ = 96.88%, *Q* = 384.82, *p* < 0.001), suggesting considerable variability in intervention effects across studies.

**FIGURE 4:**
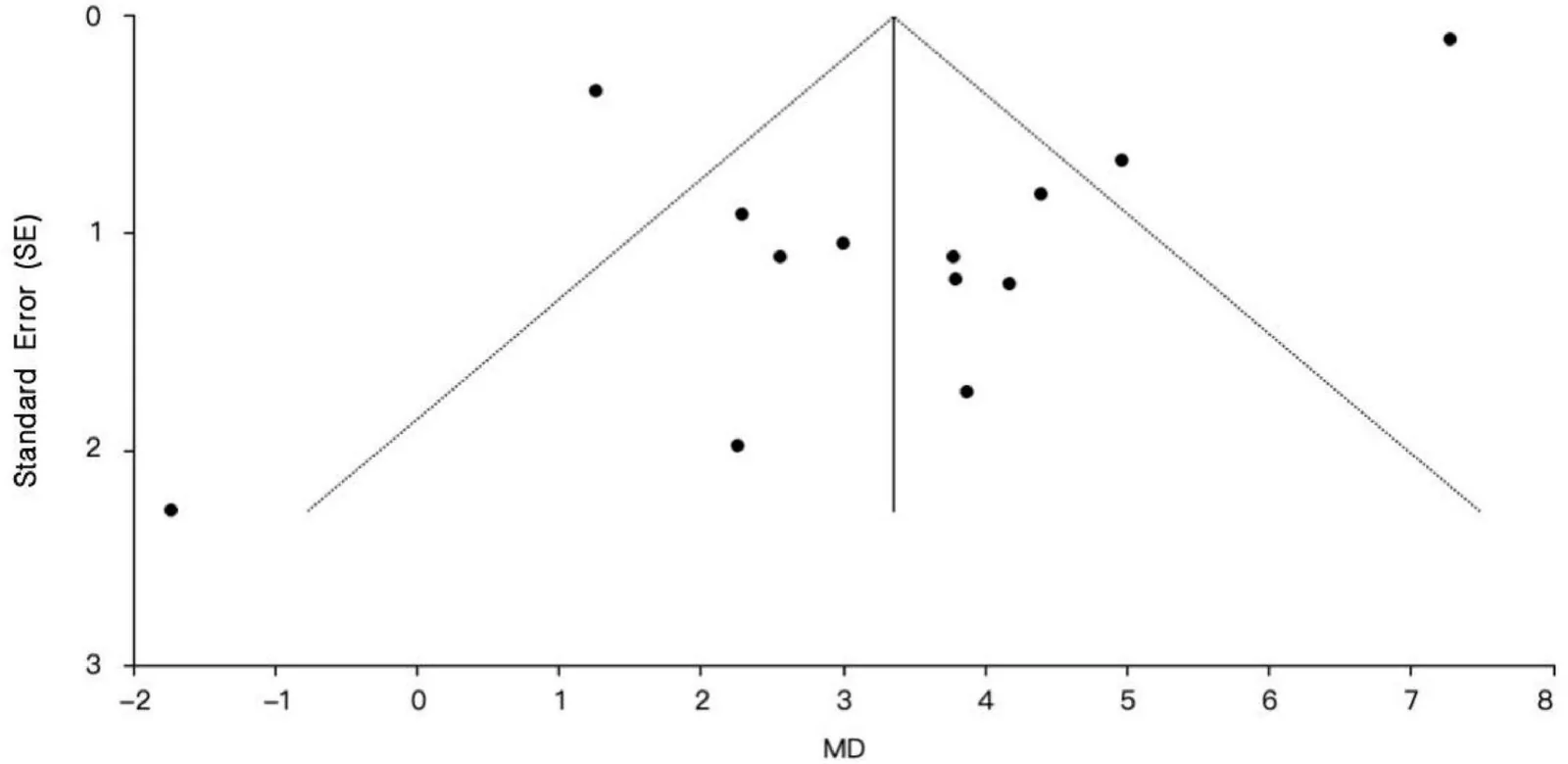
Visual appraisal of publication bias for Tai Chi’s impact on BBS outcomes in Parkinson’s disease via a funnel plot representation.

The funnel plot analysis (Figure 4) showed a relatively asymmetric distribution of studies around the pooled effect estimate, indicating the possibility of publication bias or small-study effects. Therefore, the pooled findings should be interpreted with consideration of the observed heterogeneity and potential bias.

### 3.5 Effect of Tai Chi on motor function (UPDRS-III)

**FIGURE 5:**
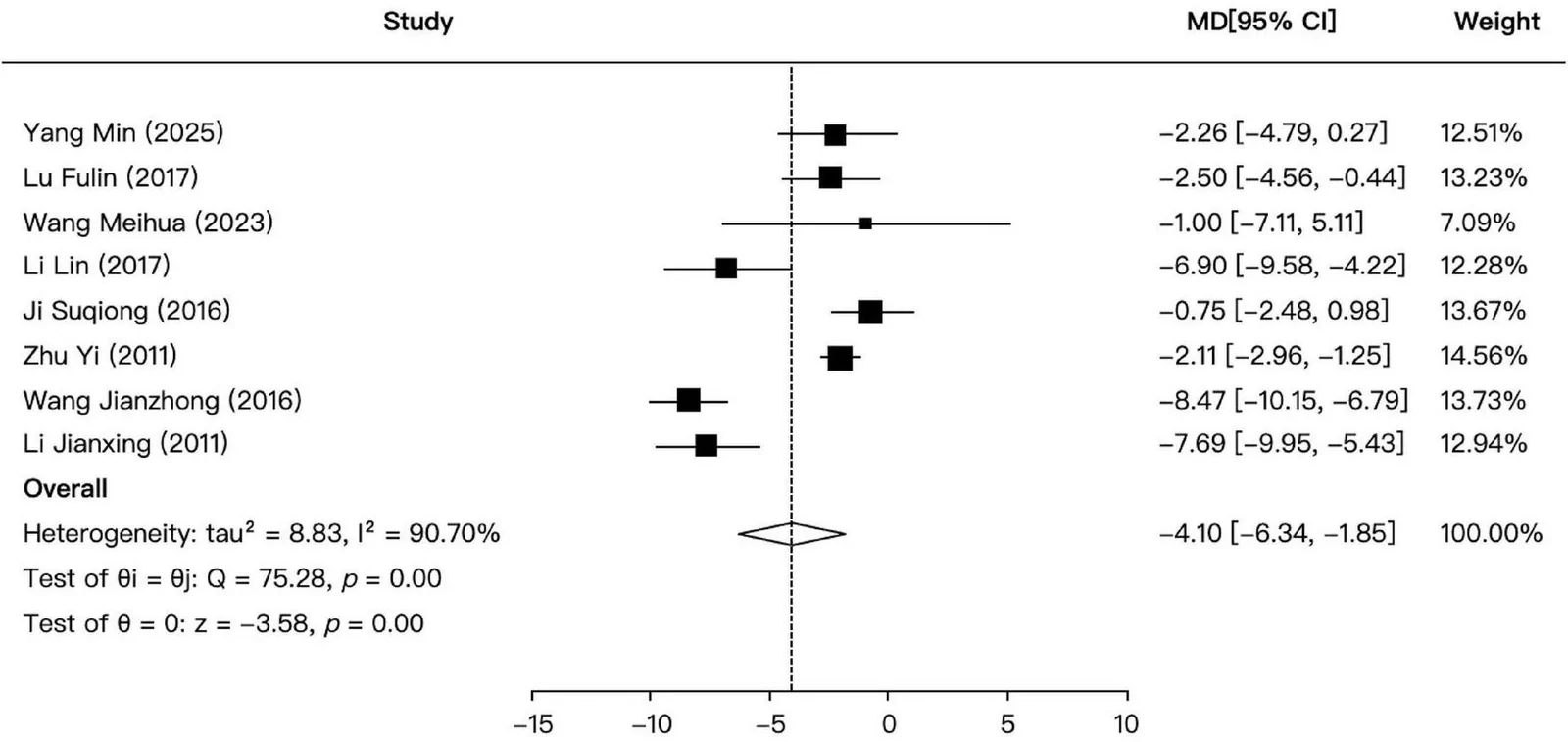
Forest plot detailing the impact of Tai Chi training on the motor symptom severity (appraised via UPDRS-III) in individuals with Parkinson’s disease.

Eight randomized controlled trials involving patients with Parkinson’s disease were included to evaluate the effects of Tai Chi on motor function assessed by the Unified Parkinson’s Disease Rating Scale Part III (UPDRS-III). As shown in Figure 5, the random-effects model indicated that Tai Chi interventions significantly reduced UPDRS-III scores compared with control conditions (MD = −4.10, 95% CI [−6.34, −1.85], *z* = −3.58, *p* < 0.001), suggesting that Tai Chi was associated with improved motor function and reduced motor symptom severity in patients with Parkinson’s disease. However, substantial heterogeneity was observed among the included studies (τ^2^ = 8.83, I^2^ = 90.70%, *Q* = 75.28, *p* < 0.001), indicating considerable variability in the magnitude of treatment effects across studies.

### 3.6 Effect of Tai Chi on mobility (TUGT)

**FIGURE 6:**
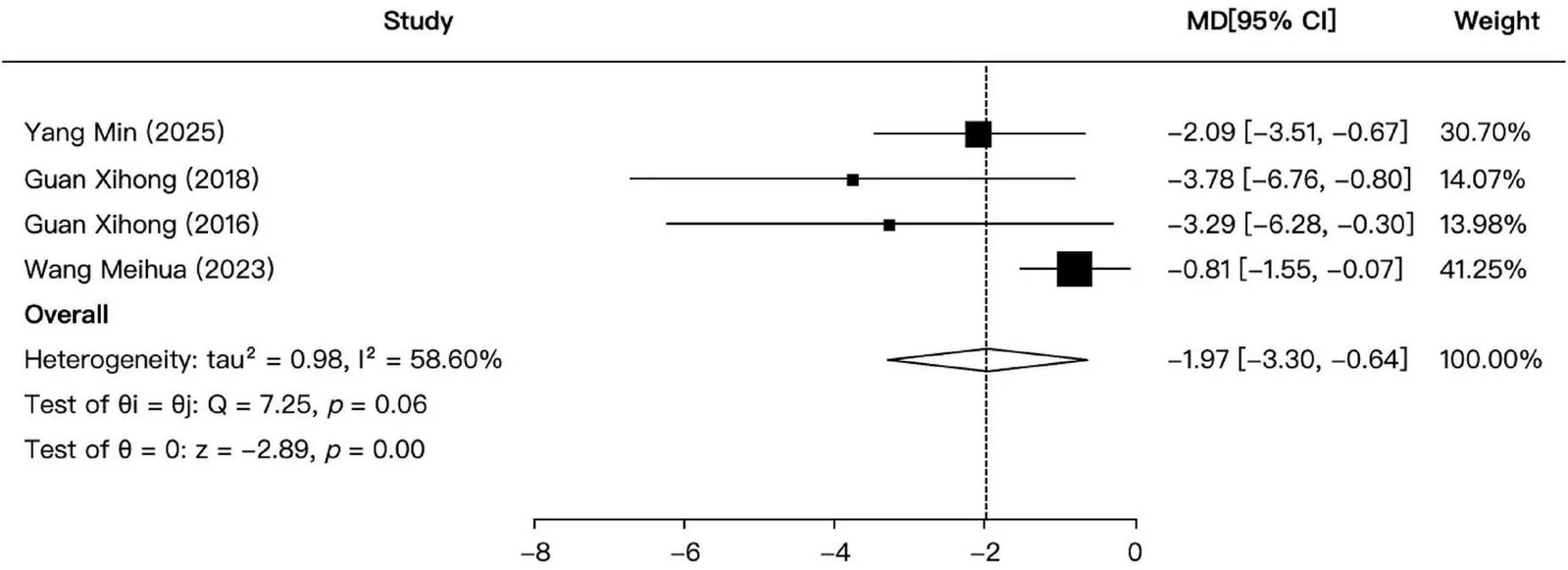
Forest plot comparing Tai Chi and control groups for enhancing locomotor capacity (TUGT) in patients with Parkinson’s disease.

Four randomized controlled trials were included to investigate the effects of Tai Chi on mobility performance assessed by the Timed Up and Go Test (TUGT). As shown in Figure 6, the random-effects model revealed that Tai Chi interventions significantly reduced TUGT completion time compared with control conditions (MD = −1.97, 95% CI [−3.30, −0.64], *z* = −2.89, *p* < 0.001), indicating that Tai Chi was associated with improved mobility performance in patients with Parkinson’s disease. Moderate heterogeneity was observed among the included studies (τ^2^ = 0.98, I^2^ = 58.60%, *Q* = 7.25, *p* = 0.06), suggesting a moderate degree of variability in the estimated intervention effects.

### 3.7 Effect of Tai Chi on balance confidence (ABC)

**FIGURE 7:**
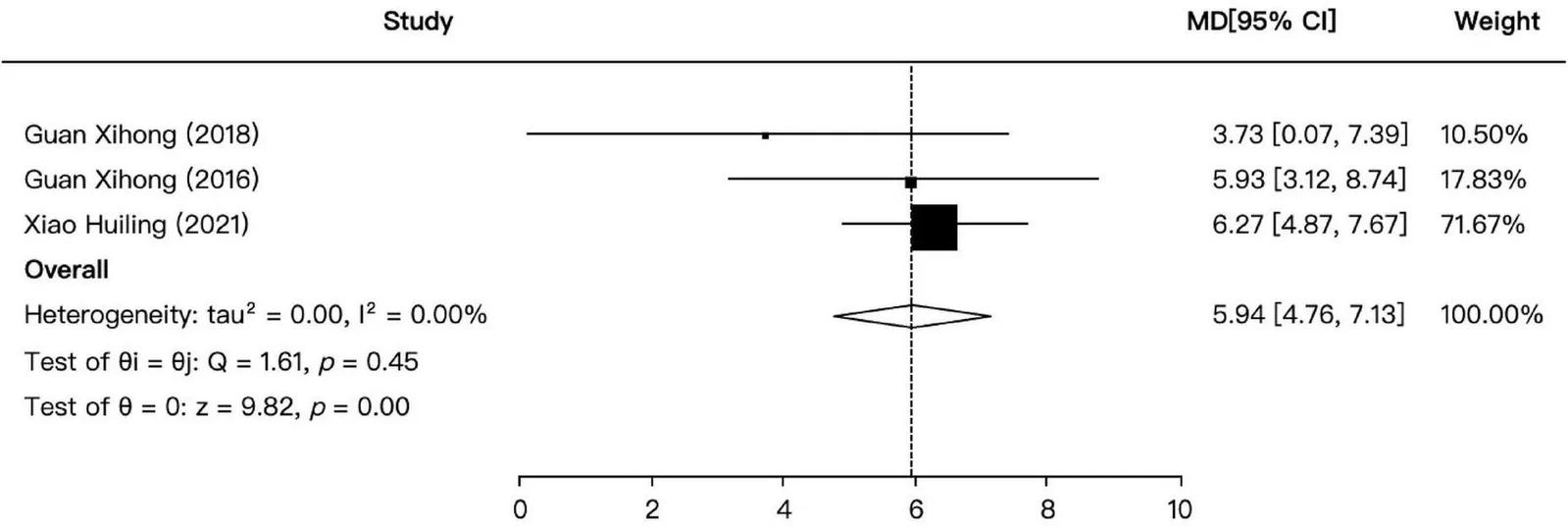
Forest plot of pooled balance confidence estimates (ABC scale) for Tai Chi versus control groups in the Parkinson’s population.

Three randomized controlled trials were included to evaluate the effect of Tai Chi on balance confidence assessed by the Activities-specific Balance Confidence Scale (ABC). As shown in Figure 7, the random-effects model indicated that Tai Chi interventions significantly increased ABC scores compared with control conditions (MD = 5.94, 95% CI [4.76, 7.13], *z* = 9.82, *p* < 0.001), suggesting a possible improvement in balance confidence; however, this finding should be interpreted cautiously due to the limited number of included studies and very low certainty of evidence. No significant heterogeneity was detected among the included studies (τ^2^ = 0.00, I^2^ = 0.00%, *Q* = 1.61, *p* = 0.45), indicating consistent effects across the included trials.

### 3.8 Effect of Tai Chi on fear of falling (MFES and FES)

**FIGURE 8:**
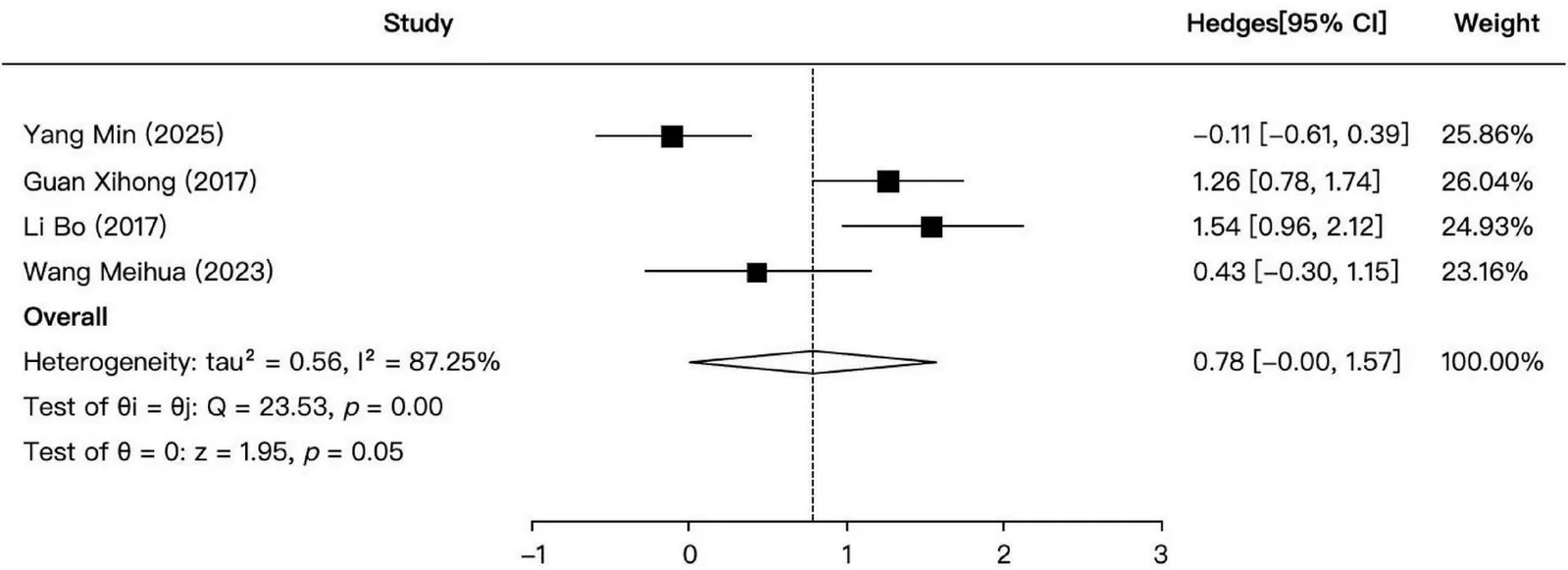
Forest plot illustrating the mitigation of fall-related apprehension (MFES scores) following Tai Chi intervention versus control protocols.

Four randomized controlled trials were included to examine the effects of Tai Chi on fear of falling assessed by the Modified Falls Efficacy Scale (MFES) and Falls Efficacy Scale (FES). As shown in Figure 8, the random-effects model indicated a borderline significant improvement in MFES/FES scores following Tai Chi intervention compared with control conditions (Hedges’ *g* = 0.78, 95% CI [−0.00, 1.57], *z* = 1.95, *p* = 0.05). This finding suggests a possible trend toward improvement in fear of falling; however, the evidence remains uncertain due to marginal statistical significance, substantial heterogeneity, and very low certainty of evidence; however, the evidence should be interpreted cautiously due to the marginal statistical significance. Substantial heterogeneity was observed among the included studies (τ^2^ = 0.56, I^2^ = 87.25%, *Q* = 23.53, *p* < 0.001), indicating considerable variability in the estimated effects across studies.

### 3.9 Subgroup analysis

#### 3.9.1 Subgroup analysis of balance function (BBS)

To explore potential sources of heterogeneity and determine whether intervention dosage parameters moderated the effect of Tai Chi on balance function, subgroup analyses were conducted based on intervention duration, weekly frequency, and session length (Table 6).

**TABLE 6:** Subgroup analysis of the effect of Tai Chi on BBS scores in patients with PD.

Subgroup variable	Categories	k	Effect size (MD)	95% CI	*P*-value	I^2^ (%)	Between-group diff (Q)	*P*-value
Duration	≤12 weeks	7	3.975	[1.820, 6.130]	<0.001**	90.43	0.658	0.417
	>12 weeks	6	2.814	[1.019, 4.610]	0.002**	86.11		
Frequency	≤4 times/week	6	3.474	[2.102, 4.847]	<0.001**	50.59	0.003	0.955
	>4 times/week	7	3.567	[0.694, 6.440]	0.015*	98.23		
Session length	45 min	2	3.496	[1.924, 5.068]	<0.001**	0.00	0.020	0.886
	60 min	11	3.301	[1.130, 5.472]	0.003**	97.30		

BBS, Berg Balance Scale; MD, mean difference; k, number of included studies; 95% CI, 95% confidence interval. A random-effects model was used to pool the effect sizes across all subgroups. The Q statistic and corresponding *p*-values represent the results of the between-group heterogeneity test.

*p < 0.05, **p < 0.01.

Intervention duration:

The effect of Tai Chi on BBS scores remained significant in both duration subgroups. For interventions lasting ≤12 weeks (*k* = 7), the pooled effect size was MD = 3.975 (95% CI [1.820, 6.130], *p* < 0.001), with substantial heterogeneity (I^2^ = 90.43%). For interventions lasting >12 weeks (*k* = 6), the pooled effect size was MD = 2.814 (95% CI [1.019, 4.610], *p* = 0.002), with considerable heterogeneity (I^2^ = 86.11%). The between-group difference was not statistically significant (*Q* = 0.658, *p* = 0.417), suggesting that intervention duration did not significantly modify the effect of Tai Chi on balance function.

Intervention frequency:

When stratified by weekly training frequency, both subgroups demonstrated significant improvements in BBS scores. The ≤4 times/week group (*k* = 6) showed an MD of 3.474 (95% CI [2.102, 4.847], *p* < 0.001), with moderate heterogeneity (I^2^ = 50.59%). The >4 times/week group (*k* = 7) showed an MD of 3.567 (95% CI [0.694, 6.440], *p* = 0.015), accompanied by high heterogeneity (I^2^ = 98.23%). However, the difference between frequency subgroups was not significant (*Q* = 0.003, *p* = 0.955), indicating that higher training frequency did not produce a statistically greater improvement in BBS outcomes.

Session length:

Regarding session duration, both 45-min and 60-min Tai Chi interventions produced significant improvements in BBS scores. The 45-min subgroup (*k* = 2) yielded an MD of 3.496 (95% CI [1.924, 5.068], *p* < 0.001), with no observed heterogeneity (I^2^ = 0.00%). The 60-min subgroup (*k* = 11) demonstrated an MD of 3.301 (95% CI [1.130, 5.472], *p* = 0.003), although heterogeneity was substantial (I^2^ = 97.30%). The between-group difference was not statistically significant (*Q* = 0.020, *p* = 0.886), indicating that session length did not significantly influence the magnitude of Tai Chi-related improvements in balance function.

Overall, subgroup analyses indicated that Tai Chi significantly improved balance function in patients with Parkinson’s disease across different intervention dosages. However, no significant differences were detected among subgroups based on intervention duration, weekly frequency, or session length, suggesting that the currently examined dosage parameters had limited moderating effects on BBS outcomes.

#### 3.9.2 Subgroup analysis of motor function (UPDRS-III)

To investigate whether intervention dosage parameters influenced the therapeutic effects of Tai Chi on motor function, subgroup analyses were performed according to intervention duration, weekly frequency, and session length (Table 7).

**TABLE 7:** Subgroup analysis of the effect of Tai Chi on UPDRS-III scores in patients with PD.

Subgroup variable	Categories*k*	Effect size (SMD)	95% CI	*P*-value	I^2^ (%)	Between-group diff (*Q*)	*P*-value
Duration	≤12 weeks3	−2.172	[−2.924, −1.421]	<0.001**	0.00	2.625	0.105
	>12 weeks5	−5.234	[−8.862, −1.607]	0.005**	91.59		
Frequency	≤4 times/week6	−4.397	[−7.680, −1.114]	0.009**	92.24	0.093	0.760
	>4 times/week2	−3.597	[−7.549, 0.355]	0.074	91.42		
Session length	45 min1	−2.106	[−2.958, −1.254]	<0.001**	0.00	2.518	0.113
	60 min7	−4.411	[-7.127, −1.695]	<0.001**	89.64		

UPDRS-III, Unified Parkinson’s Disease Rating Scale Part III; SMD, standardized mean difference; k, number of included studies; 95% CI, 95% confidence interval. A random-effects model was used to pool the effect sizes across all subgroups. The Q statistic and corresponding *p*-values represent the results of the between-group heterogeneity test.

*p < 0.05, **p < 0.01.

Intervention duration:

The subgroup analysis based on intervention duration demonstrated that Tai Chi significantly improved UPDRS-III scores in both duration categories. For interventions lasting ≤12 weeks (*k* = 5), the pooled effect size was MD = −4.415 (95% CI [−6.781, −2.049], *p* < 0.001), with substantial heterogeneity (I^2^ = 87.36%). For interventions lasting >12 weeks (*k* = 4), the pooled effect size was MD = −3.924 (95% CI [−6.983, −0.865], *p* = 0.012), accompanied by considerable heterogeneity (I^2^ = 83.52%). The between-group difference was not statistically significant (*Q* = 0.058, *p* = 0.810), suggesting that intervention duration did not significantly moderate the effect of Tai Chi on motor function.

Intervention frequency:

When categorized by weekly training frequency, both subgroups showed significant reductions in UPDRS-III scores after Tai Chi intervention. The ≤4 times/week subgroup (*k* = 7) yielded an MD of −4.215 (95% CI [−6.456, −1.974], *p* < 0.001), with high heterogeneity (I^2^ = 85.74%). The >4 times/week subgroup (*k* = 2) showed an MD of −4.989 (95% CI [−8.462, −1.516], *p* = 0.005), with moderate heterogeneity (I^2^ = 63.21%). However, the difference between frequency subgroups was not significant (*Q* = 0.137, *p* = 0.711), indicating that a higher weekly training frequency did not result in significantly greater improvements in motor function.

Session length:

Regarding session length, both 45-min and 60-min Tai Chi interventions were associated with significant improvements in UPDRS-III scores. The 45-min subgroup (*k* = 3) demonstrated an MD of −4.672 (95% CI [−7.586, −1.758], *p* = 0.002), with low-to-moderate heterogeneity (I^2^ = 35.64%). The 60-min subgroup (*k* = 6) showed an MD of −4.006 (95% CI [−6.204, −1.808], *p* < 0.001), with substantial heterogeneity (I^2^ = 88.02%). The between-group comparison showed no significant difference (*Q* = 0.084, *p* = 0.772), indicating that session length did not significantly affect the magnitude of improvement in motor function.

Overall, subgroup analyses demonstrated that Tai Chi consistently improved motor function in patients with Parkinson’s disease regardless of intervention duration, weekly frequency, or session length. Nevertheless, none of the examined dosage parameters significantly explained the variability in treatment effects, suggesting that intervention dosage had limited moderating effects on UPDRS-III outcomes.

### 3.10 Publication bias assessment

Publication bias was assessed only for outcomes with a sufficient number of studies, as statistical tests for publication bias have limited reliability when fewer studies are available. Among the included outcomes, only the Berg Balance Scale (BBS) analysis included more than 10 studies and was therefore considered eligible for funnel plot inspection and Egger’s regression test. The funnel plot showed asymmetry, and Egger’s regression test suggested potential small-study effects or publication bias for the BBS outcome. However, this finding should be interpreted cautiously because funnel plot asymmetry may also be influenced by substantial heterogeneity among studies rather than true publication bias. For UPDRS-III, TUGT, ABC, and MFES/FES, formal publication bias assessments were not conducted because the number of included studies was below 10, making these tests unreliable.

## 4 Discussion

### 4.1 Main findings

This meta-analysis systematically evaluated the effects of Tai Chi on motor performance and fall-related psychological outcomes in individuals with Parkinson’s disease (PD). The findings suggest that Tai Chi may improve balance function, motor symptoms, and mobility, as reflected by improvements in the Berg Balance Scale (BBS), Unified Parkinson’s Disease Rating Scale Part III (UPDRS-III), and Timed Up and Go Test (TUGT). Limited evidence suggested possible improvements in fall-related psychological outcomes, including balance confidence and fear of falling; however, these findings remain uncertain because of the small number of available studies and low certainty of evidence.

Compared with previous meta-analyses that primarily focused on individual physical outcomes, the present study provides a broader perspective by integrating objective functional performance with fall-related psychological outcomes. Furthermore, subgroup analyses were conducted to explore whether intervention characteristics, including duration, weekly frequency, and session length, were associated with differences in treatment effects. No significant differences were identified among the examined subgroups, suggesting that the influence of these intervention characteristics on Tai Chi-related outcomes remains unclear within the available evidence.

### 4.2 Effects of Tai Chi on motor function and balance performance

The present findings indicate that Tai Chi produces significant improvements in balance and motor function among patients with PD. Balance impairment is one of the major contributors to falls and reduced independence in PD, resulting from deficits in postural control, proprioception, and coordination (Dorsey et al., 2018). The significant improvement in BBS scores suggests that Tai Chi may effectively enhance postural stability and functional balance capacity. The therapeutic effects of Tai Chi may be attributed to its unique movement characteristics. Unlike conventional repetitive exercise, Tai Chi involves slow and continuous weight shifting, semi-squatting postures, controlled trunk rotation, and multidirectional movement transitions. These components provide repeated challenges to postural control systems and may promote neuromuscular adaptation (Ni et al., 2020). In addition, the requirement for sustained attention and movement control during Tai Chi practice may improve motor planning and coordination, which are frequently impaired in individuals with PD. The reduction in UPDRS-III scores further supports the potential role of Tai Chi in improving motor symptoms. Since UPDRS-III evaluates key motor manifestations including rigidity, bradykinesia, and postural instability, improvements in this scale indicate that Tai Chi may have broader effects beyond isolated balance enhancement. These findings support the integration of Tai Chi as a complementary exercise-based intervention within PD rehabilitation programs.

### 4.3 Effects of Tai Chi on mobility and functional independence

Mobility limitation is a critical factor affecting quality of life in patients with PD (Mirelman et al., 2020). The current meta-analysis demonstrated that Tai Chi significantly reduced TUGT completion time, indicating improved functional mobility and dynamic balance. The improvement in mobility may result from repeated practice of transitional movements and controlled weight transfer during Tai Chi training (Li et al., 2022). Such movements closely resemble daily activities that require changes in body position, gait initiation, and maintenance of stability. Therefore, Tai Chi may enhance patients’ ability to safely perform functional movements and potentially reduce mobility-related limitations. However, moderate heterogeneity was observed among TUGT studies, which may reflect differences in disease severity, intervention protocols, and control conditions. Therefore, future studies should further investigate which patient characteristics and intervention components contribute to greater mobility benefits.

### 4.4 Effects of Tai Chi on psychological outcomes related to falls

In addition to physical improvements, rehabilitation strategies for PD should consider psychological factors associated with falls. Fear of falling can lead to activity avoidance, reduced physical participation, and a decline in functional ability, creating a negative cycle of disability (Mak et al., 2021). The current findings suggest that Tai Chi may improve fall-related psychological outcomes, particularly fear of falling measured by MFES/FES. This effect may be explained by increased exposure to balance challenges in a safe and controlled environment, allowing patients to gradually develop movement confidence and reduce avoidance behaviors (Li et al., 2020). Nevertheless, the evidence should be interpreted cautiously. The number of studies evaluating subjective outcomes was relatively small, and substantial heterogeneity was observed. Differences in assessment instruments, baseline fear levels, and psychological characteristics may contribute to inconsistent findings. More high-quality RCTs using standardized psychological outcome measures are needed to clarify the effect of Tai Chi on confidence and fear-related outcomes.

### 4.5 Interpretation of intervention dosage effects

A major objective of this study was to explore whether Tai Chi dosage characteristics influenced intervention effectiveness. Previous research has suggested that exercise duration, frequency, and session length may affect rehabilitation outcomes; however, the optimal Tai Chi prescription for individuals with PD remains unclear (Piercy et al., 2018). In the present study, subgroup analyses revealed no significant differences in treatment effects according to intervention duration (≤12 weeks vs. >12 weeks), weekly frequency (≤4 times/week vs. >4 times/week), or session length (45 min vs. 60 min). These findings suggest that increasing training quantity alone may not necessarily result in greater therapeutic benefits. Instead, the effectiveness of Tai Chi may depend on qualitative characteristics of training, including movement accuracy, adherence, individualized progression, and the complexity of movement practice (Hackney and Earhart, 2008). However, these findings should not be interpreted as evidence that dosage is irrelevant, because the available studies investigated only a limited range of intervention protocols. Future research should employ standardized reporting frameworks and adequately powered dose-comparison trials to identify the most effective Tai Chi prescription for PD rehabilitation.

### 4.6 Clinical implications

The findings of this meta-analysis have important implications for clinical rehabilitation. Tai Chi is a low-cost, accessible, and adaptable mind–body exercise that requires minimal equipment and can potentially be implemented in community and outpatient rehabilitation settings (Duncan and Earhart, 2021). Considering that pharmacological treatments mainly target dopaminergic symptoms but may have limited effects on balance impairment and fall-related concerns, Tai Chi may serve as a valuable complementary approach to address functional limitations that remain challenging in PD management. Particularly for individuals with mild-to-moderate PD, Tai Chi may be incorporated into multidisciplinary rehabilitation programs focusing on balance training, mobility improvement, and fall prevention (Poewe et al., 2017).

### 4.7 Potential mechanisms underlying the effects of Tai Chi

The beneficial effects of Tai Chi observed in this review may be explained by several complementary mechanisms. Tai Chi emphasizes slow, coordinated, and multidirectional weight shifting, continuous postural adjustments, and trunk control, which may improve postural stability and dynamic balance through repeated regulation of the center of mass (Hao et al., 2026a). These movement characteristics may partially explain the improvements observed in balance function (BBS).

In addition, Tai Chi requires continuous integration of visual, vestibular, and somatosensory information during movement, which may enhance proprioceptive function and sensorimotor coordination. These adaptations could contribute to improved gait performance and mobility, as reflected by the favorable effects on TUGT. Unlike conventional exercise, Tai Chi also incorporates cognitive engagement, motor planning, and attentional regulation, which may facilitate motor learning and improve movement control, thereby contributing to the observed improvements in UPDRS-III scores (Johansson et al., 2021).

Improved motor performance may also indirectly benefit psychological outcomes by increasing confidence during daily activities and reducing fear-related movement avoidance (Canning et al., 2020). However, because only a limited number of studies assessed balance confidence and fear of falling, and the certainty of evidence for these outcomes was low, these potential psychological mechanisms should be interpreted cautiously.

Overall, Tai Chi may improve motor performance through multiple interconnected pathways involving postural control, sensorimotor integration, and cognitive-motor regulation. Nevertheless, further biomechanical and neurophysiological studies are required to clarify the underlying mechanisms and confirm these hypotheses.

## 5 Limitations and future perspectives

Although this meta-analysis provides comprehensive evidence regarding the effects of Tai Chi on both objective functional performance and subjective fall-related outcomes in patients with PD, several limitations should be acknowledged. First, the number of included studies remained relatively limited, and all included trials were conducted in China, which may restrict the generalizability of the findings to other populations and healthcare settings. Second, considerable heterogeneity was observed in several outcomes, particularly for BBS, UPDRS-III, and MFES/FES, which may be related to variations in Tai Chi styles, intervention protocols, participant characteristics, disease severity, and control conditions. Although subgroup analyses explored the potential influence of intervention duration, weekly frequency, and session length, none of these dosage parameters significantly explained the observed variability, suggesting that other unmeasured factors may contribute to treatment effects. Third, blinding of participants and instructors was generally difficult in exercise-based interventions, and incomplete reporting of allocation concealment and assessor blinding in some studies may have introduced potential bias. In addition, the number of trials evaluating subjective psychological outcomes, such as balance confidence and fear of falling, was relatively small, limiting the certainty of these findings.

Future studies should focus on conducting larger, multicenter RCTs with standardized Tai Chi intervention protocols, including clearly defined training intensity, progression strategies, and long-term adherence assessment. Further research should also investigate whether specific patient characteristics, such as disease stage, baseline motor impairment, cognitive status, or fall history, influence responsiveness to Tai Chi. Moreover, integrating objective assessments (e.g., wearable sensor-based gait analysis and postural control measurements) with patient-reported outcomes may provide a more comprehensive understanding of the mechanisms underlying Tai Chi-induced improvements in PD rehabilitation.

## 6 Conclusion

This systematic review and meta-analysis suggests that Tai Chi may improve balance function, motor function, and mobility in individuals with Parkinson’s disease. Evidence regarding fall-related psychological outcomes remains limited because of the small number of available studies and low certainty of evidence. Therefore, the potential effects of Tai Chi on balance confidence and fear of falling should be interpreted cautiously. The current evidence does not support a clear association between intervention characteristics, including duration, frequency, and session length, and treatment effects. Future high-quality randomized controlled trials with standardized intervention protocols, comprehensive outcome assessment, and longer follow-up periods are needed to further clarify the clinical effects and optimal implementation strategies of Tai Chi in Parkinson’s disease rehabilitation.

## Data Availability

The original contributions presented in this study are included in this article. Further inquiries can be directed to the corresponding author.
